# Life Events, Coping, and Posttraumatic Stress Symptoms among Chinese Adolescents Exposed to 2008 Wenchuan Earthquake, China

**DOI:** 10.1371/journal.pone.0029404

**Published:** 2012-01-25

**Authors:** Yuhong Zheng, Fang Fan, Xianchen Liu, Lei Mo

**Affiliations:** 1 Center for Studies of Psychological Application, South China Normal University, Guangzhou, China; 2 Department of Psychiatry, Indiana University, Indianapolis, Indiana, United States of America; University of Sydney, Australia

## Abstract

**Purpose:**

To examine the relationship between negative life events, coping styles, and symptoms of post-traumatic stress disorder (PTSD) among adolescent survivors exposed to 2008 Wenchuan Earthquake, China.

**Methods:**

A survey was conducted in a sample of 2250 adolescent students from two schools in Dujiangyan District, a seriously damaged area, 20 kilometers away from the epicenter, 6 months after the earthquake. Participants completed a self-administered questionnaire including demographics, negative life events, coping styles, and PTSD symptoms.

**Results:**

Academic pressure was the strongest predictor of adolescents' PTSD symptoms among all negative life events. Main effects of negative life events, positive coping and negative coping on PTSD symptoms were significant in both younger adolescents and older adolescents, while the moderator effects of two coping styles were found significant only within older adolescents.

**Conclusions:**

Coping may play a role to moderate the relationship between post-earthquake negative life events and PTSD symptom, but the function seems to depend on the age of participants. Psychosocial coping skills training may be important in the prevention and intervention of mental health problems in adolescent survivors of traumatic earthquake.

## Introduction

A strong earthquake, with a Richter-scale magnitude of 8.0, hit Wenchuan County in Sichuan Province at 14∶28 on May 12, 2008. The nation's worst earthquake in three decades caused enormous casualties. According to the State Council Information Office, the death toll from the quake in the affected regions reached 69,227, and the number of people listed as missing and injured were 17,923 and 374,643, respectively (official figures from http://www.scio.gov.cn/gzdt/ldhd/200809/t222722.htm). It was estimated that approximately one million children and adolescents were affected by the devastating earthquake, including those who were displaced, transferred, physically injured, lost family members or lost personal belongings.

A number of studies have examined the association between natural disasters and Post-Traumatic Stress Disorder (PTSD). In a review of 225 studies [1], 166 studies reported that natural disasters were associated with elevated risks of PTSD or related symptoms. In the past few decades, there has been increasing concern regarding earthquake-related PTSD in juvenile population. For instance, Giannopoulou et al. [2] found that the prevalence of PTSD 6–7 months after 1999 Athens earthquake was 35.7% among youhts aged 9 to 17. Hsu et al. [3] reported that 21.7% of 323 school students aged 12 to 14 had PTSD symptoms 6 weeks after the 1999 earthquake in Taiwan. Zhao et al. [4] reported that the incidence of PTSD was 9.4% among adolescent victims 17 months after the 1998 Zhangbei earthquake in China. The variability of PTSD rates across studies could be attributed to differences in the nature and severity of the earthquake, the timing of the psychiatric assessment, and the instruments used in the study.

According to a conceptual model regarding predictors for PTSD in children [5], the incidence and severity of PTSD are associated with the child's exposure to the disaster, pre-disaster individual's demographics (e.g. gender, age, race), characteristics of the social environment and children's coping. For example, researchers have found a dose-response relationship between earthquake exposure and PTSD symptoms in children [6]. More specifically, symptoms of PTSD were more frequently reported among those who perceived life threat [2], lost family members [3], and had severely house damaged [7]. With regard to gender, females often reported higher level of severity of PTSD than males, and this conclusion is not limited to youths but also adults [8], [9]. Findings of age difference on PTSD among children and adolescents are inconsistent across studies. Giannopoulou et al. [2] found that the 9–11-year-old group reported more PTSD symptoms than the 12–14 years old, and the 12–14-year-old group had more PTSD symptoms than the 15–17-year-old group. Whereas, Bal and Jensen [10] reported no significant difference on PTSD scores among students aged 8 to 15. In a previous epidemiological research on the current sample, Fan et al. [11] reported that older adolescents (aged 15–18) had higher prevalence of PTSD symptoms than the younger group (aged 11–14).

In addition to the trauma exposure itself, life disruption following the traumatic event has been found to be associated with the incidence and severity of PTSD in a body of literature. Evidence from adult-based research suggests that post-disaster negative life events, including loss of job or income, broken relationships, serious illnesses or injuries in the victims and death or illness in close acquaintances, were significantly associated with PTSD [12], [13]. However, little has been done to examine the effect of post-trauma life stressors on PTSD symptoms among children and adolescents. One of the few studies found that post disaster disruption of family life increased the risk for psychopathology than the traumatic event itself among school-aged children exposed to a brush fire in Australia [14]. It is worth pointing out that everyday life stressor and its meaning for individuals may differ across subjects with different cultural backgrounds. In China, for example, middle school and high school students suffer from extreme academic pressure, not only because of the limited opportunities to get into higher education, but also because of the high expectations from their parents. In this study, we sought to examine whether the ongoing daily life stressors (e.g., academic pressure) following a disaster would contribute to Chinese youngsters' PTSD symptoms.

Coping refers to active efforts to master, reduce, or tolerate the demands created by stress [15], involving a series of cognitive and behavioral strategies. Although previous studies have not reached agreement on the categories of coping styles [16], some researchers suggest classifying coping on the basis of the aims and intentions of different coping behaviors [17]. In light of the purposes of different behaviors, youths' coping can be divided into 2 types: approach coping and avoidant coping [17]. Approach coping that aims to directly address the stressor includes behaviors such as problem-solving, seeking social support and cognitive reconstructing. By contrast, avoidant coping refers to behaviors that aim to avoid the problematic situations, including denial, blaming, social withdrawal and disengagement. Similarly, coping in Chinese adolescents is classified by researchers into two categories: positive coping and negative coping [18], with the former comparable to approach coping and the later comparable to avoidant coping. A large body of literature has shown that both coping styles are associated with mental health problems [19]. In post-disaster studies, researchers have found that coping is an important predictor of psychological symptoms. For example, Vernberg et al. [5] noted that more frequent use of positive coping, blame and anger, as well as social withdrawal were associated with increased PTSD symptoms in children after Hurricane Andrew. Vernberg et al. argued that the positive relationship between positive coping and PTSD symptoms, which seems inconsistent with a large body of disaster-related literature, may be due to the fact that children were processing the traumatic experience [5]. However, positive coping was found to be a strong protective factor for PTSD symptoms in many post-disaster studies [20], suggesting that positive coping is functional in ameliorating PTSD symptomatology. On the other hand, findings from empirical studies have shown that avoidant coping has a negative impact on posttraumatic symptoms of adolescent victims after hurricanes [21] or earthquakes [22], and even significantly predicted medium and long term PTSD symptoms [23].

In addition to directly ameliorating or deteriorating psychological distress, coping has been found to moderate the relationship between stressors and mental health outcomes in a few of empirical researches [24], [25]. For example, findings from a recent study showed that avoidant coping strengthened the association between traumatic experience and PTSD symptoms [25]. Luthar et al. [26] noted that it would be useful to conceptualize the protective and vulnerability process as more complex interactive processes. In present study, we tried to explore the moderator effects of types of coping on the relationship between post-earthquake negative life events and PTSD symptoms among Chinese adolescents.

To sum up, the purposes of this study were 1) to examine the impact of different types of post-earthquake negative life events on adolescents' PTSD symptoms, and 2) to explore the moderating effect of either positive coping or negative coping on the relationship between post-earthquake negative life events and PTSD symptoms. In addition, given that previous researches have suggested that younger adolescents may differ from older adolescents in symptoms of PTSD [2], [10] as well as the utilization of coping strategies [27], [28], the moderation of coping would be tested based on developmental level.

## Methods

### Ethics Statement

The study was approved by Human Research Ethics Committee of South China Normal University. Informed written consent from participants' parents was obtained before the assessment, with oral assent from the participants. Participants were also told that they could choose not to answer any questions in the questionnaires and they were free to withdraw from the study at any time during data collection.

### Subjects

Participants were sampled from one middle school (grades 7–9) and one high school (grades 10–12) in Dujiangyan District, a seriously damaged area located 20 kilometers away from the epicenter, with 3069 people killed, 4388 injured as well as 429 missing . The two schools were selected for three reasons: 1) they were public schools with a large number of enrollments, 2) students' characteristics were comparable to all students in the local area, and 3) the school principals were willing to participate in the study. We did not select schools in the epicenter (Wenchuan County), because most of those schools were destroyed and the students were transferred to schools outside the county. On the contrary, schools in Dujiangyan District were not seriously destroyed, though this area was also directly hit by the quake. Students from grades 7–10 were chosen for this study, because this was a longitudinal project and participants could be followed for at least 2 years before they graduated from their current schools. Finally, the convenience sample consisted of 2463 students from grades 7–9 in the middle school and grade 10 in the high school, and 2250 of them returned their questionnaires.

### Procedure

With permission and support from the school boards and Chengdu Women Federation, we collected data in November of 2008, approximately 6 months after the disaster. Participants were asked to complete a couple of self-administered questionnaires in pencil-and-paper format. Questions included demographics, earthquake exposure, negative life events, social support, coping styles, resilience, anxiety, depression and PTSD. Participants completed their questionnaires in class with the help of well-trained interviewers who were psychological professionals from South China Normal University and had experiences with administering mental health and psychological tests. The entire assessment took approximately 40 minutes for the participants to complete. Finally, it should be noted that data reported here were the first wave of our longitudinal research program. An epidemiological report of the same sample has been published elsewhere [11]. A media report of the current program can be found at http://www.bjreview.com.cn/Post_Wenchuan/2009-05/27/content_241985_2.htm.

### Measures

#### Demographics

Demographic items included age, gender and family residence. Participants were divided into younger adolescents group (11–14 years old, recoded as 0) and older adolescents group (15–18 years old, recoded as 1). Gender was recoded as 0 for male, and 1 for female. Residence was recoded as 0 for urban, and 1 for rural.

#### Earthquake Exposure

Four questions were used to evaluate respondents' earthquake exposures: 1) Did you have a close family member dead or seriously injured? 2) To what extent was your house damaged? 3) To what extent was your property (other than house) loss? and 4) Did you directly witness the tragic disaster? Then 4 items were generated based on the responses to the questions, including casualties of family members (0 = no, 1 = injured, 2 = killed/missing), house damage (0 = no, 1 = moderately damaged, 2 = severely damaged), property loss (0 = no, 1 = moderate loss, 2 = severe loss), and directly witness (0 = no, 1 = yes). These 4 items would be used as individual items in the data analysis.

#### Post-earthquake negative life events

Post-earthquake negative life events were evaluated by the Adolescent Self-Rating Life Events Checklist (ASLEC) [29]. It consists of 26 items, including 6 dimensions: interpersonal conflicts, academic pressure, being punished, personal loss, physical health problems, and others. The impact of each negative life event over the past 6 months was rated on a 5-point Likert scale, from 1 = none to 5 = very severe. All the 26 items were added up to generate a total score. The ASLEC has been used for a number of studies [30], [31] and has acceptable psychometric properties [29]. In this sample, Cronbach's alpha for internal consistency was .88.

#### PTSD

The Posttraumatic Stress Disorder Self-Rating Scale (PTSD-SS) [32] was used to assess the severity of PTSD symptoms. The PTSD-SS was developed on the basis of the DSM-IV diagnostic criteria of PTSD [33] and the Chinese Classification of Mental Disorders [34]. It contains 24 items to measure the impact of a traumatic event (i.e., Wenchuan Earthquake) over the previous 6 months on a 5-point scale, from 1 = none to 5 = very severe. Total PTSD scores were calculated by summing scores across all items. A score of 50 or higher was used to classify a respondent as having symptoms of PTSD [32]. The psychometric properties of PTSD-SS have been previously described in a Chinese sample [32]. In the present sample, Cronbach's alpha for the internal consistency was .94.

#### Coping Style

The Simplified Coping Style Questionnaire (SCSQ) [35] was used to measure coping styles. It is a 20-item scale with two subscales: positive coping and negative coping. The positive coping subscale consists of 12 items, which refer to behaviors that actively buffer the frustrated situation, such as ‘trying to find effective resolutions’. On the other hand, negative coping is evaluated by 8 items that refer to avoidant behaviors, such as ‘through smoking, drinking or using substances to get relief. Respondents were asked to rate each item on a 4-point scale to indicate how often they used a particular strategy to deal with a problem or stressful situation, from never = 0 to often = 3. Subscale scores were generated by averaging scores for the items on each subscale, with higher scores indicating more frequent use of the coping style. Good reliability has been reported in previous study [35]. In this study, the subscales have acceptable internal consistencies, with Cronbach's alpha being .79 for positive coping and .66 for negative coping.

### Statistical analysis

A set of independent t-test were performed to compare the difference between younger adolescents and older adolescents on negative life events, coping and symptoms of PTSD. Pearson correlations were used to describe the relationships between negative life events, coping styles and PTSD symptoms. Multiple regression analysis was used to examine the effect of negative life events on PTSD symptoms. Hierarchical regression analysis was performed to examine the moderating effect of coping styles with negative life events on PTSD symptoms. Age group, gender, residence and earthquake exposure were included as covariates for multiple analysis because our previous report showed that all of them were significantly associated with post-traumatic symptoms [11]. All of the analyses were conducted with SPSS 13.0 for Windows. The threshold of statistical significance was set at *p*<.05.

## Results

### Participant Characteristics

Of 2250 participants, 2069 (91.9%) completed the measures of negative life events, coping, and PTSD. The sample consisted of 1119 (54.1%) females with a mean age of 14.56 (*SD* = 1.31) and 950 (45.9%) males with a mean age of 14.61 (*SD* = 1.35); 1508 (76.4%) were urban residents and 489 (23.6%) were rural residents.

### Negative life events, coping and PTSD symptoms among different age groups

As shown in Table 1, older adolescents reported higher score on negative life events (*df* = 2067, *p*<.001) as well as PTSD symptoms (*df* = 2067, *p*<.001) than the younger group. Younger adolescents had higher scores on positive coping than the older group (*df* = 2067, *p*<.05), while no significant difference on negative coping was found between the two age groups (*df* = 2067, *p*>.05).

**Table 1:** Scores of Negative Life Events, Coping and PTSD Symptoms among Different Adolescent Group.

	Younger adolescents	Older adolescents	
	n = 824	n = 1245	
	*Mean (SD)*	*Mean (SD)*	*t*
Negative life events	41.70 (10.63)	47.88 (13.39)	11.12***
Positive coping	1.76 (.54)	1.71 (.49)	2.40*
Negative coping	1.21 (.56)	1.25 (.51)	1.83
PTSD symptoms	34.63 (11.35)	39.21 (15.13)	7.41***

*p<.05,

***p<.001.

### Correlations between negative life events, coping, and PTSD symptoms

Table 2 presents means, standard deviations, and bivariate correlations of negative life events, PTSD symptoms, and 2 coping styles. As shown in Table 2, all life events subscales and negative coping were positively related to PTSD symptoms, but positive coping was negatively correlated with PTSD symptoms (all *p* values<.001). All life events subscales and negative coping were significantly and positively related to each other (all *p* values<.001). Positive coping was negatively related to all life events subscales (all *p* values<.01) except personal loss.

**Table 2:** Bivariate Correlation Among Life Events, Coping, and PTSD Symptoms (N = 2069).

	*Mean*	*SD*	1	2	3	4	5	6	7	8	9
1. PTSD symptoms	37.39	13.92									
2. Total ASLEC score	45.42	12.73	.48***								
3. Interpersonal conflicts	8.71	2.94	.36***	.81***							
4. Academic pressure	10.95	3.58	.46***	.79***	.61***						
5. Being punished	10.04	3.85	.29***	.80***	.61***	.55***					
6. Personal loss	5.15	2.66	.29***	.67***	.45***	.37***	.47***				
7. Physical health problems	5.84	2.03	.34***	.63***	.38***	.42***	.37***	.39***			
8. ‘Others’	5.84	2.24	.31***	.71***	.53***	.45***	.66***	.34***	.40***		
9. Positive coping	1.73	0.51	−.14***	−.12***	−.10***	−.13***	−.06**	−.01	−.05*	−.15***	
10. Negative coping	1.24	0.53	.22***	.26***	.23***	.21***	.18***	.13***	.16***	.24***	.24***

*p<.05;

**p<.01;

***p<.001.

### Effects of negative life events on PTSD symptoms

A multiple regression analysis was performed to examine the impact of each type of negative life event on PTSD symptoms, with gender, age group, residence, casualties of family members, house damage, property loss and witness as covariates. As shown in Table 3, after controlling the effect of demographics and earthquake exposures which accounted for 10.8% for the variance, academic pressure was the strongest predictor of PTSD symptoms among all life events, accounting for 15.4% of the total variance. The second significant predictor of PTSD symptoms was physical health problems, which accounted for 2.2% of the variance. Interpersonal conflicts, being punished, and ‘others’ together contributed to 1.1% of the total variance. Personal loss did not enter the regression model. Being punished was negatively but slightly associated with PTSD symptoms.

**Table 3:** Multiple Regression Analysis: Negative Life Events and PTSD Symptoms (N = 2069).

	*B*	*SE*	*β*	*R^2^*
Covariates				.108
Gender	2.04***	.53	.07***	
Age group	.56	.59	.02	
Family residence	1.73**	.66	.05**	
Casualties of family members	2.92***	.37	.15***	
House damage	−.03	.45	−.01	
Property loss	1.12*	.53	.05*	
Witness of tragic disaster	3.25***	.53	.12***	
Negative life events				
Interpersonal conflicts	.41**	.12	.09**	.003
Academic pressure	1.14***	.10	.29***	.154
Being punished	−.21*	.10	−.06*	.001
Physical health problems	.93***	.15	.14***	.022
‘Others’	.64***	.16	.10***	.007

Note: For entire model, *F*(12, 2056) = 72.66^***^, Adjusted *R^2^* = .294.

*p<.05.

**p<.01.

***p<.001.

### Moderating Analysis

Hierarchical multiple regression analyses were performed to examine the moderating effect of positive and negative coping in the relationship between negative life events and PTSD symptoms, respectively. According to the procedure recommended by Frazier et al. [36], the independent variable (total score of negative life events) and the moderator variables (positive coping and negative coping) were centralized before generating the interaction terms in order to eliminate multicollinearity. For both hierarchical models, demographics and earthquake exposures were entered in Step 1 as covariates. Negative life events and the 2 coping styles were entered in Step 2. Finally, the two-way interaction terms (Negative Life Events×Positive Coping and Negative Life Events×Negative Coping) were entered in Step 3. Two separate hierarchical multiple regression analyses were conducted, one for each of the two age groups (Table 4).

**Table 4:** Hierarchical Multiple Regression Analysis: Negative Life Events, Coping and PTSD Symptoms.

Dependent Variable = PTSD	*B*	*SE*	*β*	*R^2^*	*ΔR^2^*	Δ*F(dfs)*
Younger adolescents (age <15)						
Step 1				.059	.059	8.60***(6, 817)
Gender	1.21	.72	.05			
Family residence	−1.41	1.73	−.03			
Casualties of family members	2.23	.52	.14***			
House damage	.13	.58	.01			
Properties loss	1.03	.71	.05			
Witness of the tragic disaster	1.44	.73	.06*			
Step 2				.215	.155	53.70***(3, 814)
Negative life events (NLE)	.34	.04	.32***			
Positive coping	−2.76	.71	−.13***			
Negative coping	2.79	.69	.14***			
Step 3				.217	.002	1.13(2, 812)
NLE×Positive coping	−.09	.06	−.05			
NLE×Negative coping	.07	.06	.04			
Older adolescents (age ≥15)						
Step 1				.110	.110	25.40***(6, 1238)
Gender	2.99	.72	.10***			
Family residence	2.67	.74	.09***			
Casualties of family members	3.09	.50	.14***			
House damage	−.32	.64	−.04			
Properties loss	1.66	.75	.06*			
Witness of the tragic disaster	4.51	.73	.15***			
Step 2				.324	.214	129.88***(3, 1235)
NLE	.40	.03	.35***			
Positive coping	−.36	.75	−.12***			
Negative coping	4.63	.74	.16***			
Step 3				.338	.014	13.40***(2, 1233)
NLE×Positive coping	−.19	.05	−.09***			
NLE×Negative coping	.21	.05	.10***			

Note.

*p<.05.

**p<.01.

***p<.001.

Negative life events, positive coping and negative coping were centralized before generating the interaction terms.

For the younger adolescents, the main effects of negative life events, positive coping as well as negative coping on PTSD symptoms were statistically significant. However, neither of the two-way interactions significantly predicted PTSD symptoms.

**Figure 1:**
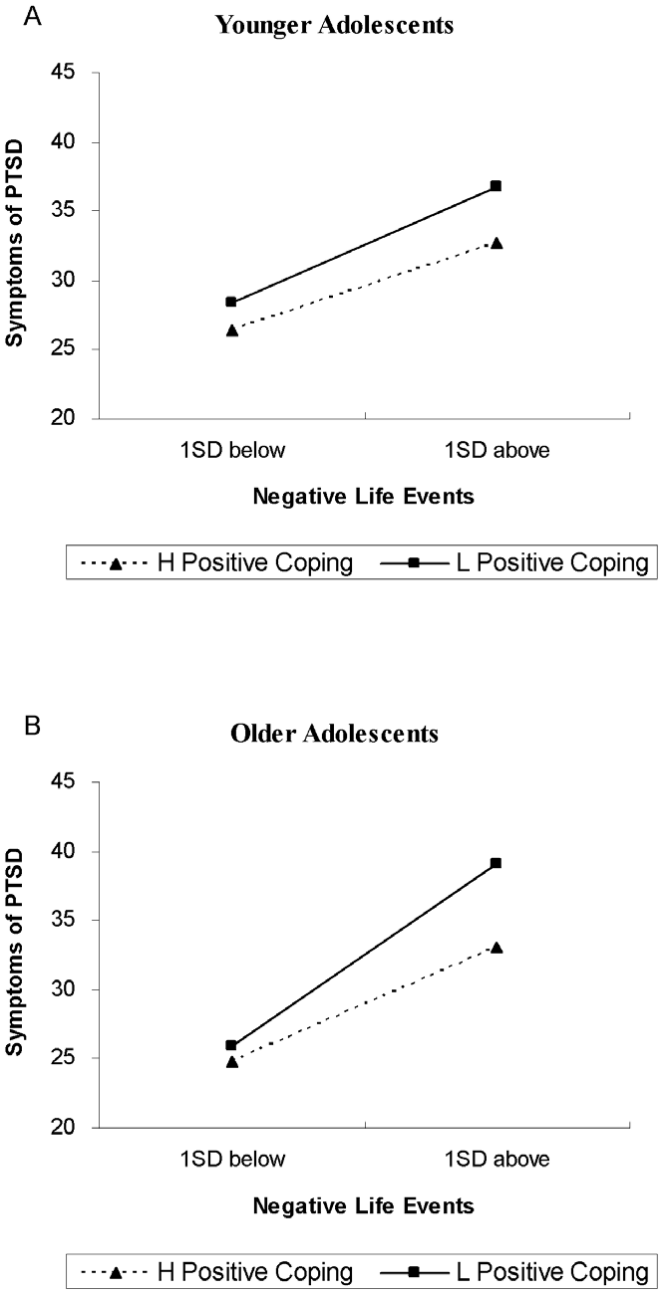
Interaction of positive coping and negative life events on PTSD symptoms for younger adolescents and older adolescents. This figure reveals the moderation of positive coping in the association between negative life events and PTSD symptoms among two age groups. For younger adolescents, the interaction of positive coping and negative life events is not statistically significant. For older adolescents, however, a significant interaction of positive coping and negative life events is found. Specifically, the relationship between negative life events and PTSD symptoms is stronger among those with low levels of positive coping than those with high levels of positive coping. As negative life events increase, participants with low levels of positive coping will have more PTSD symptoms than those with high levels of positive coping.

**Figure 2:**
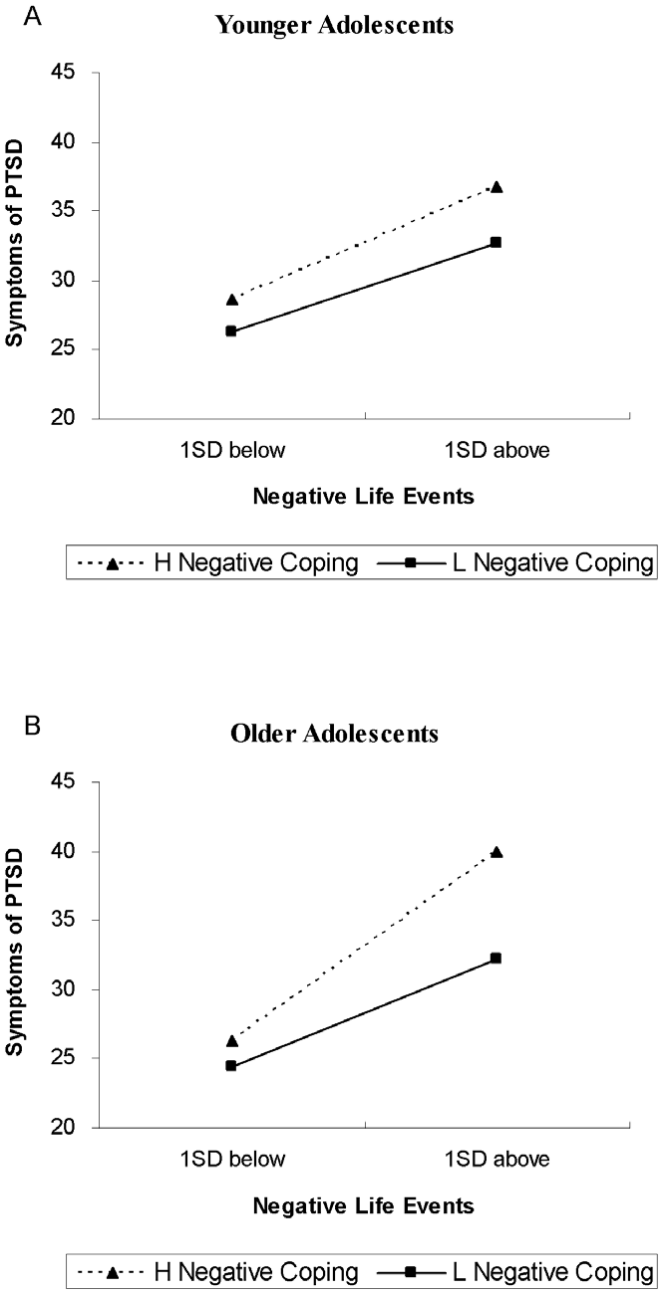
Interaction of negative coping and negative life events on PTSD symptoms for younger adolescents and older adolescents. This figure reveals the moderation of negative coping in the association between negative life events and PTSD symptoms among two age groups. For younger adolescents, the interaction of negative coping and negative life events is not statistically significant. For older adolescents, however, a significant interaction of negative coping and negative life events is found. Specifically, the relationship of negative life events and PTSD symptoms is stronger among those with high levels of negative coping than those with low levels of negative coping. As negative life events increase, participants with high levels of negative coping will have more PTSD symptoms than those with low levels of negative coping.

For the older adolescents, results showed that negative life events, positive coping and negative coping significantly predicted PTSD symptoms. In addition, the regression coefficients for the 2 two-way interaction terms were statistically significant, indicating the relationship between negative life events and PTSD symptoms was moderated by both positive and negative coping. Simple effect of negative life events at two levels of the moderator was tested by simple slope analyses [37]. As shown in Figure 1B, the association between negative life events and PTSD symptoms was significant at both low and high levels of positive coping (*B*_low_ = .49, *p*<.001; *B*_high_ = .30, *p*<.001). Nevertheless, results indicated that the relationship between negative life events and PTSD symptoms was stronger among those with low levels of positive coping than those with high levels of positive coping. Similarly, as depicted in Figure 2B, the relationship between negative life events and PTSD symptoms was significant at both low and high levels of negative coping (*B*_low_ = .29, *p*<.001; *B*_high_ = .50, *p*<.001). However, results indicated that the association between negative life events and PTSD symptoms was stronger among those with high levels of negative coping than those with low levels of negative coping.

## Discussion

The present study aimed to examine the impact of post-earthquake negative life events on PTSD symptoms and to explore the role of coping styles in the relation between negative life events and PTSD symptoms among adolescent survivors of the 2008 Wenchuan earthquake in China. Results showed that negative life events were significantly associated with PTSD symptoms after controlling for demographics and earthquake exposure. Among various post-disaster negative life events, academic pressure was most strongly associated with PTSD symptoms. Both positive and negative coping moderated the relationship between negative life events and PTSD symptoms in adolescents aged 15 to 18 years old, while the interaction of negative life events and coping did not predict PTSD symptoms in younger adolescents aged 11–14 years old.

The finding that post-disaster negative life events were related to PTSD symptoms was consistent with previous studies [14], [38], suggesting that ongoing life disruption following trauma is a potential risk for victim's psychopathology. Our results revealed that academic pressure was the main subsequent stressor contributing to PTSD symptoms among Chinese adolescent survivors. As a major life stressor for Chinese adolescents, academic pressure has been found to be associated with mental health problems in prior work [31], [39], [40]. Given that there is comparatively limited access to higher education for an enormous number of Chinese students, it makes a great deal of sense that students in China may encounter great pressure from schools, which poses a potential threat to adolescents' mental health, particularly to those who had experienced a devastating earthquake. Another type of negative life event strongly and positively associated with PTSD symptoms was physical health problems. This finding corresponded to a large body of literature noticing a significant link between physical and mental health distress among people exposed to trauma [9], [41]. Interpersonal conflicts and personal loss were also significantly associated with PTSD symptoms, supporting previous reports that PTSD symptoms were more severe among traumatized individuals who had poor interpersonal relationships [12], had loss of property [42], or loss of work following disasters [13].

It should be noted that being punished was associated with decreased PTSD symptoms after adjustment for covariates and other life event subscales. This finding was contrary to our expectation, because being punished is a life stressor that is hypothesized to be positively related to PTSD symptoms as other negative life events are. Zhao and Zhao found that being punished before the disaster was negatively associated with post-earthquake psychological symptoms [43], but the association between being punished after the earthquake and PTSD symptoms has not been reported in previous studies. However, it is worth noticing that the association was not highly significant (*β* = −.06, *ΔR* ^2^ = .001) in present study. Further research is warranted to examine if this association is true or is due to chance or measurement bias, such as the multicollinearity between being punished and other negative life events.

Positive and negative coping were both significantly associated with PTSD symptoms among our subjects, which is globally consistent with a large body of literature that shows that more frequent use of positive, problem-focused coping was protective for the posttraumatic symptoms [20], [44] and that more frequently using negative, avoidance-focused coping was a risk for posttraumatic symptoms [21], [45]. However, results revealed that positive coping and negative coping significantly moderated the relationship between negative life events and PTSD symptoms only within older adolescents. For youths aged 15 to 18, those who tended to adopt more positive coping strategies had lower severity of PTSD symptoms, and those who tended to use negative coping strategies were more vulnerable to PTSD symptoms. These findings may be attributed to the fact that older adolescents may have better cognitive and emotional capacity to process the ongoing stressful experience than younger adolescents. Higher positive coping is more protective for older adolescents than younger adolescents, which slows the increase in PTSD symptoms as negative life events accumulate. On the other hand, higher negative coping is a risk for the older group and accelerates the increase in PTSD symptoms when negative life events increase.

The study has several limitations. To begin with, despite we measured participants' negative life events during the 6 months following the earthquake, there might be some bias because the data was obtained in a retrospective self-report approach. Yet, for ethnical reasons, we could not administrate the assessment until half year after the disaster. Second, as noted above, the participants in this study were a convenient sample from one middle school and one high school in Dujiangyan. It is unknown if those findings from the convenient sample could be generalized to other adolescent survivors. Third, the study was cross-sectional, making it impossible to conclude a causal relationship between life events, coping, and PTSD symptoms. Since this is a longitudinal study, our further analysis with follow up assessments would help clarify causal relationships.

In conclusion, this is the first study that examines the associations of post-disaster life events and coping with PTSD symptoms in Chinese adolescents after the 2008 Wenchuan earthquake. The present study extends existing literature in the field of life stress, coping, and PTSD with a large sample of Chinese adolescent survivors of an earthquake. This study demonstrated the impact of post-earthquake life events on PTSD symptoms and the moderating effect of coping on the relationship between life events and PTSD symptoms. These findings have important implications in the prevention and intervention of post-traumatic mental health problems in adolescent survivors. First, since academic pressure may be a risk for adolescents' post-disaster symptoms, teachers and parents should be aware of the responsibility to create supportive recovery environments for adolescents, and to shift their expectations of adolescents' school achievement. Second, it is imperative to develop active problem-solving skills and strategies for traumatized youths to deal with stressful life situations following disasters and diminish post-traumatic reactions and psychopathology.
